# Development and Preliminary Validation of a Scale to Measure Perceived Therapist’s Knowledge about Gender Identity Diversity among Trans and Non-Binary Individuals in Puerto Rico

**DOI:** 10.3390/ejihpe14070125

**Published:** 2024-06-25

**Authors:** Caleb Esteban, Eddiel Hernández-López, Margarita Francia-Martínez, Alixida Ramos-Pibernus

**Affiliations:** 1 The Queer Biopsychosocial Health Laboratory (The Queer Lab), School of Behavioral and Brain Sciences, Ponce Health Sciences University, Ponce 00716, Puerto Rico; edhernandez21@stu.psm.edu; 2 Ponce Research Institute, Ponce Health Sciences University, Ponce 00716, Puerto Rico; 3 Sex, Gender, and Sexual Orientation Diversity Committee, Puerto Rico Psychological Association, San Juan 00936, Puerto Rico; mfrancia@albizu.edu (M.F.-M.); aliramos@psm.edu (A.R.-P.); 4 Clinical Psychology Program, Universidad Carlos Albizu, San Juan 00901, Puerto Rico; 5 Health Equity Research (HER) Lab, School of Behavioral and Brain Sciences, Ponce Health Sciences University, Ponce 00716, Puerto Rico

**Keywords:** trans/transgender, gender non-binary, gender minorities, knowledge, scale development and validation

## Abstract

This study aimed to describe the development of the Perceived Therapist’s Knowledge about Gender Identity Diversity Scale and to preliminarily validate this scale by describing its psychometric properties. This research instrument was constructed based on the existing literature and recommendations for instrument development. Initially, a 36-item scale was devised to assess perceived openness and knowledge about gender identity diversity in therapy. The content validation process involved 12 expert judges, leading to a refined 25-item scale. Participants consisting of 57 trans and non-binary Puerto Rican individuals completed the scale. Exploratory factor analysis revealed a unidimensional structure, supporting a single factor named “perceived knowledge about gender identity diversity in therapy.” The final scale demonstrated excellent reliability (α = 0.978; *S*α = 0.980; ω = 0.979), indicating strong internal consistency. This validated scale contributes to assessing primarily Hispanic trans and non-binary individuals’ perceptions of their therapists’ knowledge about gender identity diversity.

## 1. Introduction

The LGBTQ+ community, particularly gender minorities or individuals identifying as trans and gender non-binary (TGNB), has historically faced pervasive stigmatization, discrimination, and prejudice [1,2,3]. This discrimination extends across cultural, legal, institutional, and religious contexts. Even within therapeutic settings, TGNB individuals encounter challenges, ranging from subtle biases, such as microaggressions, to total discrimination [3,4]. There is an urgent need to assess and improve the knowledge of mental health professionals regarding gender identity diversity in therapy spaces.

A previous study conducted in Puerto Rico where therapists were surveyed [5] found that 14.9% had moderately prejudiced attitudes toward trans individuals who undergo a social transition and 19.8% had these attitudes toward trans individuals who undergo a physical transition. In addition, 50% of the sample reported having moderate social distance toward trans individuals. These results make visible the existing prejudice fostered by therapists on the island towards trans people and create an opportunity for action.

The TGNB population often experiences discrimination from both heterosexual individuals and sexual minorities (lesbian, gay, and plurisexual) [1,3]. Therefore, receiving therapy from a sexual minority would not necessarily exempt the client or patient from prejudices or discrimination or imply better or adequate service. In addition, TGNB individuals may not even perceive affirmative care in specialized affirmative-gender clinics [6]. Prejudices and discrimination have been related to a lack of knowledge about gender identity and other factors [7,8]. Research indicates that biases among mental health professionals, including counselors and psychologists, also persist [1]. These biases manifest in subtle ways, such as comments reinforcing stereotypes or questioning the legitimacy of gender identities. However, studies by McCann and Sharek [9,10] emphasize the importance of therapists’ cultural competence, their ability to establish a positive therapeutic alliance, and their attitudes towards clients/patients’ sexual orientations and gender identities. Therefore, there is a need to understand and document the experiences of TGNB individuals in therapy spaces concerning their perception of the therapist’s knowledge regarding gender identity diversity.

Several scales have been identified that measure attitudes and knowledge toward TGNB individuals, such as the Transgender Attitudes and Beliefs Scale [11], the Transsexual Prejudice Scale [12], the Attitudes Toward Transgendered Individuals Scale [13], the Transprejudice Scale [14], the Transphobia Scale [15], the Genderism and Transphobia Scale [16], the Negative Attitudes towards Trans People Scale [17], Attitudes toward Trans Men and Women [18], Perceptions of Providers’ Cultural Competency [19], and the Transgender Health Care Humanization Scale [20]. Although these instruments have been widely utilized to address attitudes and knowledge regarding the TGNB community, they collect information from the health provider’s perspective and not the client/patient.

However, two instruments, published after our study, have been found that measure topics related to TGNB attitudes of openness and knowledge from the client/patient’s perspective. The Trans-Inclusive Provider Scale [21] is a 27-item, self-response 5-point rating scale, ranging from (1) not important to (5) very important, covering six dimensions: trans-inclusive messaging, name and pronoun usage, outreach, gender-affirming practice, referral comfort, and inclusive intake forms. This scale measures inclusive behavioral indicators toward TGNB individuals in general healthcare settings. More recently, a survey was conducted to measure patients’ perceived level of clinician knowledge of transgender healthcare [22]. This measure consists of three questions: (1) “Thinking about the doctor or provider you go to for your trans-related healthcare (such as hormone treatment), how much do they know about providing healthcare for trans people?”; (2) “Do you see your trans-related provider for routine care?”; and (3) “How much does your routine healthcare provider (who you see for physicals, flu, diabetes, etc.) know about healthcare for trans people?” (p. 4). This instrument uses a self-response 5-point rating scale, ranging from (1) knows almost everything to (5) I am not sure. Yet, this is a composite measure of questions and not a developed and validated instrument. Although our study’s instrument measures similar constructs, it mainly focuses on mental health professionals and emphasizes more objective trans-affirmative recommended and evidence-based competencies, such as openness and TGNB knowledge, that are presented during therapy.

The integration of the American Psychological Association (APA) Guidelines for Psychological Practice with Transgender and Gender Nonconforming People [23] and the version at the time (2012) of the World Professional Association for Transgender Health’s (WPATH) Standards of Care for the Health of Transsexual, Transgender, and Gender Nonconforming People (V7) [24] underscores the essential knowledge base required by therapists working with transgender and gender non-binary individuals. As a framework, it emphasizes that therapists should possess a comprehensive understanding of the biopsychosocial aspects of gender diversity and gender-affirming care. Therapists should be equipped with knowledge about culturally competent and affirmative practices in mental health, ensuring a supportive therapeutic environment for TGNB clients and patients. By integrating guidelines and standards, therapists gain insights into the evolving principles of gender-affirming interventions, such as knowledge and openness, enabling them to collaborate effectively with other professionals and provide well-informed direction to clients and patients navigating gender issues.

The development of a scale to measure the client/patient’s perception of their therapist’s openness and knowledge about gender identity diversity in therapy spaces is crucial. Studies in Puerto Rico have found that mental health providers tend to report low or moderate bias toward TGNB individuals [5]. However, when surveying TGNB individuals, they report high bias and low knowledge from their mental health providers [25]. The historical context of discrimination, existing knowledge gaps, subtle or unconscious biases, unique challenges faced by TGBN individuals, and the need for culturally competent and inclusive mental health services collectively emphasize the urgency of this tool [26]. By comprehensively assessing TGBN patients’ perceptions of their therapists’ knowledge about gender identity diversity, the scale can guide interventions to enhance cultural competence, reduce biases, reduce health disparities, and ultimately contribute to more inclusive and effective mental health care for TGNB individuals.

This study aimed to (1) describe the development of the Perceived Therapist’s Knowledge about Gender Identity Diversity Scale and(2) to preliminarily validate the scale, describing its psychometric properties.

## 2. Method

This study used secondary data [25] and followed an instrumental construction design [27]. The study surveyed TGNB individuals aiming to evaluate differences in psychotherapy service satisfaction between cisheterosexual and TGNB clients/patients and determine whether said satisfaction was related to the therapist’s knowledge and attitudes of openness toward TGNB-related issues. This study used mixed methods, with a cross-sectional non-experimental design.

### 2.1. Procedure

The main study, which includes phases one and two, was submitted for approval to the Institutional Review Board of the Albizu University, San Juan Campus (Fall 19-23). The initial phase involved creating the instrument. Initially, a comprehensive instrument was devised, drawing upon findings from existing research literature such as the American Psychological Association Guidelines for Psychological Practice with Transgender and Gender Nonconforming People [23] and The World Professional Association for Transgender Health Standards of Care for the Health of Transexual, Transgender, and Gender Nonconforming People [24] in accordance with Boateng et al.’s [27] recommendations for instrument construction. A set of 36 items was formulated to assess perceived openness to explore gender identity (14 items) and knowledge about gender identity diversity in therapy (22 items). Consequently, two dimensions were created to measure openness and knowledge. To ensure a variety of perceptions, the team incorporated a Likert-style measurement. This scale ranged from totally disagree to totally agree. Thorough scrutiny and refinement of all items were undertaken by the team, with a concerted effort to phrase them positively.

The second phase of the content validation process involved a total of 12 clinical psychologist judges and specialists in LGBT+ issues (e.g., LGBT+ topic professors, affirmative model certified, labored in LGBT+ specialized centers, members of a professional LGBT+ committee, and/or LGBT+ issues researchers). A letter of invitation together with the first version of the instrument was sent via email. The document contained the purpose of the instrument, conceptual definitions, instructions, and the 36-item scale. The items of the scale were supplemented with an essentiality option (i.e., essential, non-essential, and essential but needs modifications), and a space for suggestions and feedback at the end. The Lawshe Technique was performed [28] to carry out item rating for essentiality. Content Validity Ratio (CVR) scores ranged from 0.50 to 0.92. A total of 11 items were eliminated for scoring a CVR below 0.56 (e.g., item 3: “My therapist comprehended how the knowledge they have about gender identity could affect the quality of the therapeutic services;” item 7: “My therapist seemed comfortable talking about my gender identity;” and item 25: “My therapist understood the difference between disclosure (coming out) of the sexual orientation and the gender identity”). There were no new item suggestions. The final scale ended with a total of 25 items and maintained the two initial domains (i.e., openness (8 items) and knowledge (17 items)).

The last phase consisted of the validation process of the scale. The team used secondary data for this process. Participants in the original study had to meet the following inclusion criteria: (1) be 21 years or older (legal adulthood in Puerto Rico), (2) be a resident of Puerto Rico, (3) have received psychotherapy services, and (4) identify as trans or gender non-binary. Using the SurveyMonkey platform, anonymous participants provided written informed consent to be part of the study, and secondary data were obtained from it.

### 2.2. Participants

From the main study, the team found a total of 57 self-identified trans and non-binary clients/patients who were actively in therapy and completed the scale. The majority of the sample identified as female (31.7%%), trans (50.9%), pansexual (28.1%), with a partner(s) (47.4%), between the ages of 21 and 30 years (76.9%), no religious affiliation (70.2%), with an income less than $12,000 (70.2%), and with a bachelor’s degree (42.1%) (See Table 1). The participants reported being from 27 of the 78 different municipalities of the islands of Puerto Rico [25].

### 2.3. Instruments

#### 2.3.1. Sociodemographic Questionnaire

A sociodemographic data questionnaire was used to gather sociodemographic information. This questionnaire included questions such as age, sex (identified, not sex assigned at birth), gender identity, sexual orientation, income, religious affiliation, relationship status, and municipality of residence [25].

#### 2.3.2. Perceived Therapists’ Openness and Knowledge about Gender Identity Diversity Scale

The Perceived Openness and Knowledge about Gender Identity Diversity in Therapy Scale was developed by the team of the original study [25]. This second version of the scale included 25 items that measured the perception of trans and non-binary individuals of the openness (8 items) and knowledge (17 items) about gender identity diversity by their therapist during the therapy process. The scale had a five-point Likert-type scale with the following options: (1) totally disagreed, (2) somewhat disagreed, (3) neutral, (4) somewhat agreed, and (5) totally agreed. The scores that a person can obtain on the total scale range from 25 to 125, with a higher number indicating more perceived openness and knowledge about gender identity diversity (see Table 2 for item examples).

### 2.4. Data Analysis Plan

Descriptive and summary statistics were calculated for demographic variables and the newly formed scale. This included calculating means, standard deviations, and reliability assessments. We employed the total variable correlation index (*r_bis_*) to evaluate the discriminative potential of the variables, which needed to exceed a threshold of 0.70, as suggested by Field [29] and Hair et al. [30] for nearly 60 participants. Reliability was evaluated using both Cronbach’s alpha and McDonald’s omega coefficients, with values greater than 0.90 being deemed as high reliability and strong internal consistency according to Field [29] and Kline [31].

For the exploratory factor analysis, we used the principal axis extraction method with oblique rotation to identify the underlying latent variables of the items. This method was chosen because it does not rely on the assumption of normality, and oblique rotation provides more accurate information. The team determined the number of factors based on two criteria: the scree plot and factors explaining at least 5% of the variance. The team used IBM SPSS statistical software (v29) to conduct descriptive and summary statistics and Stata (v18) to perform the confirmatory factorial analysis using the structural equation model.

## 3. Results

**Figure 1:**
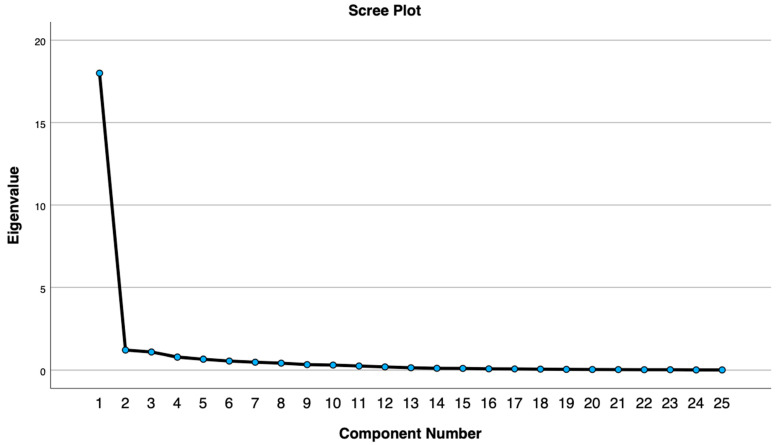
Sedimentation and variance explained by three factors.

The first exploratory factor analysis was carried out to evaluate the adequacy of the data and determine how many factors explain 5% or more of the variance to be retained. For this analysis, three components were extracted. The Kaiser–Meyer–Olkin Test supported the adequacy of the sampling data for the analysis, KMO = 0.905. Bartlett’s test of sphericity was significant (*p* < 0.001). However, considering the scree plot and that two of the components only have two items (items 8 and 10) in component 2 and one item (item 5) in component 3, and 10 items did not load on any component (items 1, 3, 6, 7, 9, 11, 12, 14, 15, and 22), the team decided to retain only one factor (see Figure 1 and Table 2).

The second analysis was performed to restrict the 15 loaded items to one factor. The Kaiser–Meyer–Olkin Test supported the adequacy of the sampling data for the analysis (KMO = 0.892) and Bartlett’s test of sphericity was significant (*p* < 0.001). Still, items 5 and 8 were not loaded in this one-factor scale. A third round was performed with 23 items, eliminating those two items (items 5 and 8). The Kaiser–Meyer–Olkin Test supported the adequacy of the sampling data for the analysis (KMO = 0.888) and Bartlett’s test of sphericity was significant (*p* < 0.001). However, this 13-item version of the scale had two items related to the openness subscale (items 2 and 4). As less than three items are not recommendable for a construct [27], the team decided to eliminate item 2 (“My therapist showed openness or interest in the struggles regarding my community’s rights”) and kept item 4 (“My therapist seemed to understand that my gender identity is not a disease”). Based on their expertise, the content of the item also measures knowledge. The team also examined the items to identify possible redundancy or duplicity in their content; however, none were found.

The last round was executed to explore the factor and discrimination analysis by calculating the item–total correlation index for this 12-item scale. The exploratory factor analysis showed a structure of one factor that explained 82.13% of the variance. The Kaiser–Meyer–Olkin Test supported the adequacy of the sampling data for the analysis (KMO = 0.885) and Bartlett’s test of sphericity was significant (*p* < 0.001). In turn, the discrimination analysis revealed that all items presented indices greater than 0.70 in the factor. Table 3 shows the factor loadings and discrimination indexes and communalities obtained by the items.

**Figure 2:**
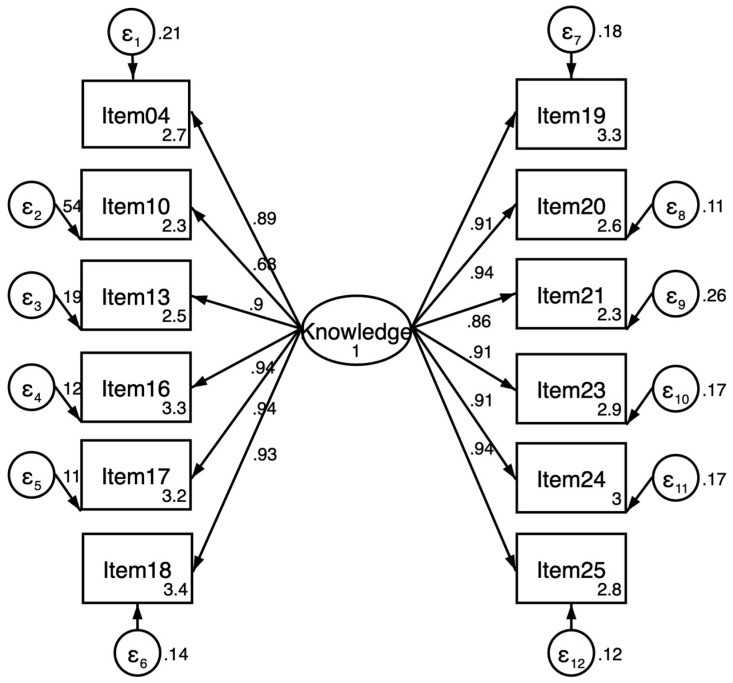
Confirmatory structural model with single factor loading.

A confirmatory factorial analysis was performed using structural equation modeling. Only the last version was evaluated using a maximum likelihood model, 95% confidence, with default standard errors. The final structural model showed good (>0.70; 11 items) and acceptable (>0.60; 1 item) Raykov’s reliability coefficients (Range = 0.68–0.94) [32] for all items (see Figure 2).

When grouping the items of the factor loading, the factor was named: perceived knowledge about gender identity diversity in therapy. The perceived openness about gender identity diversity in therapy subscale was eliminated due to the lack of statistical support. Finally, the reliability of the final scale was analyzed. For this purpose, Cronbach’s alpha, standardized Cronbach’s alpha, and McDonald’s Omega coefficients were calculated. The final scale showed excellent coefficients (α = 0.978; *S*α = 0.980; ω = 0.979).

## 4. Discussion

The purpose of this study was to describe the development and preliminary validation of a scale to measure perceived therapist openness and knowledge about gender identity diversity in therapy. However, the openness subscale was not statistically supported; therefore, the final version of the instrument only measures perceived therapist knowledge about gender identity diversity in therapy. This instrument, based on the main guidelines and standards, is the first of its kind to address and measure TGNB individuals’ perception of their mental health therapists’ knowledge concerning gender identity diversity. Since providers tend to underreport bias and possibly overreport knowledge (consciously or unconsciously) [26], there is an urgent need to survey and document clients/patients’ perceptions of their providers’ affirmative care.

As reported in the results section, the final version of this instrument consisted of 12 items (see Appendix A). The findings demonstrated that the scale has adequate psychometric properties to assess this phenomenon based on a one-factor model. Henceforth, the 12-item final version of this instrument loaded strongly on this single factor. In addition, the reliability indices and confirmatory factor analysis have shown satisfactory internal consistency and external validity for this instrument. Consequently, this instrument is recommended to be implemented for future research, specifically for the Spanish-speaking Hispanic population.

Attitudes of openness and knowledge towards TGNB individuals are two constructs highly correlated with each other in theoretical terms. Research indicates that TGNB individuals often face discrimination and a lack of understanding from mental health providers [33,34]. The retention of the knowledge factor suggests that the scale might be more effective in measuring mental health providers’ specific knowledge about gender identity diversity rather than their attitudes toward openness. The prominence of this factor suggests that mental health providers’ knowledge about gender identity may be a crucial factor in the provision of competent and inclusive care among TGNB Hispanic individuals. Thus, training and experience are key factors in improving mental health providers’ knowledge of TGNB individuals [34].

### 4.1. Strengths and Limitations

This study has several strengths that contribute to its methodological rigor: (1) it draws upon established guidelines and standards for psychological practice with TGNB individuals, (2) the involvement of 12 expert judges in the content validation process ensures the refinement and validity of the measurement tool, (3) the inclusion of 57 TNGB individuals in the sample, with diverse criteria such as age, sex, gender identity, income, and others characteristics, and (4) the instrument reported excellent reliability coefficients that instill confidence in the internal consistency of the final scale. However, several limitations must be acknowledged: (1) the cross-sectional design of the main study limits the ability to track changes over time, (2) the relatively small sample size, though reasonable since the sample was comprised of TGNB who had gone through a therapy process, might constrain the generalizability of findings, and (3) geographic limitation to Puerto Rico does not necessarily make the instrument useful for other Spanish-speaking cultures. In addition, the exclusion of the perceived openness subscale warrants further discussion regarding its implications for a comprehensive assessment of therapists’ competencies in therapy. Therefore, the development of a future scale to exclusively measure the attitudes of openness toward gender identity diversity among mental health providers is highly recommended in order to fill this gap among Hispanic gender minority individuals.

### 4.2. Future Directions

Future studies utilizing the developed instrument assessing perceptions of therapists’ knowledge about gender identity diversity in therapy could benefit from additional investigations. For example, cross-cultural validations of the scale are recommended to ensure its applicability across diverse Spanish-speaking contexts. Adaptations, translations, and additional validations are also recommended. To reinforce the validity and reliability, it is recommended to engage in longitudinal studies. Administering the instrument at various points in a therapeutic relationship and assessing the test–retest reliability would explain the stability of responses over time, offering valuable insights into how perceptions evolve. Additionally, conducting comparative studies with existing scales measuring related constructs, such as therapist empathy or cultural competence, would assess the instrument’s convergent and divergent validity. The validation of the instrument in various clinical and non-clinical settings is essential to establish its reliability across different therapeutic contexts. Further research might delve into the intersectionality of identities within this population and how these intersecting factors influence perceptions of therapists’ attributes.

### 4.3. Conclusions

In conclusion, the final version of the scale is appropriate to measure TGNB individuals’ perception of their mental health provider’s knowledge concerning gender identity diversity. The scale is designed in such a way that it is easy to comprehend, correct, and interpret. This instrument could be used to measure therapist knowledge as a protective factor or, conversely, the lack of knowledge as a risk factor for TGNB’s physical and mental health. It could also be practical for evaluation and/or intervention programs, especially to measure knowledge between groups or between times. In addition, this instrument could be implemented in future research and clinical settings for TGNB Spanish-speaking individuals.

## Figures and Tables

**Table 1:** Demographic characteristics of the sample.

Variable	*f*	*%*
Sex		
Female	18	31.7
Trans Female	12	21.1
Trans Male	12	21.1
Male	10	17.5
Non-Binary	3	5.3
Intersex	2	3.5
Gender Identity		
Trans	29	50.9
Non-Binary	26	45.6
Genderfluid	1	1.8
Orthogonal	1	1.8
Sexual Orientation		
Pansexual	16	28.1
Bisexual	13	22.8
Homosexual (gay or lesbian)	12	21.1
Heterosexual	11	19.3
Questioning	2	3.6
Asexual	1	1.8
Queer	1	1.8
Other	1	1.8
Relationship Status		
Without Partner/s	27	47.4
Living Together	15	26.8
With Partner/s But Not Living Together	9	15.8
Married	5	8.8
Widower	1	1.8
Age		
21–30	44	76.9
31–40	8	14.2
41–50	4	7.1
51–60	1	1.8
Religious Affiliation		
No	40	70.2
Yes	17	29.8
Income		
Less than $12,000	40	70.2
$12,001 to $32,000	13	22.8
$32,001 to $52,000	2	3.5
$52,001 to $72,000	1	1.8
$72,001 to $92,000	1	1.8
Education		
Less than High School	1	1.8
High School	13	22.8
Technical Degree	9	15.8
Associate Degree	2	3.5
Bachelor	24	42.1
Master	7	12.3
Doctorate	1	1.8

Note: (*n* = 57) [5].

**Table 2:** Factor loadings greater than 0.70 in the 25-item version of the scale.

Items	1	2	3
(19) My therapist was knowledgeable about the violence I might experience because of my gender identity (k)	0.892		
(18) My therapist recognized the stigma that exists about my community (k)	0.885		
(25) My therapist seemed to understand the challenges in romantic and sexual relationships that my gender identity brings (k)	0.870		
(20) My therapist recognized the institutional barriers I encounter because of my gender identity (k)	0.866		
(17) My therapist seemed to know how society’s biases affect my physical health (k)	0.854		
(23) My therapist seemed to recognize the stress that affects me from being a social minority (k)	0.830		
(16) My therapist seemed to know how society’s biases affect my mental health (k)	0.778		
(2) My therapist showed openness or interest in the struggles regarding my community’s rights (o)	0.766		
(24) My therapist was knowledgeable about how sources of support were needed to strengthen my mental health (k)	0.752		
(4) My therapist seemed to understand that my gender identity is not a disease (o)	0.722		
(13) My therapist recognized that gender identity and sexual orientation are separate concepts (k)	0.720		
(21) My therapist understood how my gender identity can vary over time (k)	0.717		
(8) My therapist informed me about their experience, education, and/or training on gender identity (o)		0.766	
(10) My therapist understood that gender is socially constructed (k)		0.758	
(5) My therapist made negative comments about my gender identity (o)			−0.797
(1) My therapist explored my gender identity (o)			
(3) My therapist showed comfort in talking about my gender identity (o)			
(6) My therapist assumed I was cisgender (o)			
(7) My therapist took into consideration the harmony between my spiritual beliefs and my gender identity (o)			
(9) My therapist understood that there are more genders than just feminine and masculine (k)			
(11) My therapist was aware that gender identity might not be aligned with sex assigned at birth (k)			
(12) My therapist recognized that gender is a continuous construct (k)			
(14) My therapist was knowledgeable about how my gender identity affects my relationship with my family (k)			
(15) My therapist understood the difference between disclosure (*coming out*) of sexual orientation and disclosure of gender identity (k)			
(22) My therapist recognized that my mental health issues were not caused by my gender identity, but by the consequences of transphobia (k)			

Note: (*n* = 57); o = openness subscale; k = knowledge subscale.

**Table 3:** Factor loading, communalities, and item means and standard deviations.

Items	*r* *_bis_*	*h* ^2^	*M*	*SD*
(20) My therapist recognized the institutional barriers I encounter because of my gender identity	0.946	0.895	3.88	1.52
(16) My therapist seemed to know how society’s biases affect my mental health	0.940	0.884	4.19	1.32
(17) My therapist seemed to know how society’s biases affect my physical health	0.937	0.878	4.00	1.49
(25) My therapist seemed to understand the challenges in romantic and sexual relationships that my gender identity brings	0.932	0.869	4.14	1.33
(18) My therapist recognized the stigma that exists about my community	0.924	0.854	4.09	1.43
(23) My therapist seemed to recognize the stress that affects me from being a social minority	0.921	0.859	4.02	1.53
(24) My therapist was knowledgeable about how sources of support were needed to strengthen my mental health	0.918	0.843	3.95	1.61
(4) My therapist seemed to understand that my gender identity is not a disease	0.918	0.843	4.25	1.29
(13) My therapist recognized that gender identity and sexual orientation are separate concepts	0.917	0.841	3.93	1.41
(19) My therapist was knowledgeable about the violence I might experience because of my gender identity	0.908	0.824	4.18	1.30
(21) My therapist understood how my gender identity can vary over time	0.873	0.762	3.60	1.58
(10) My therapist understood that gender is socially constructed	0.717	0.513	3.56	1.64

Note: (*n* = 57); *r_bis_* = Discrimination Index; *h*^2^ = Communalities; *M* = Item Mean; *SD* = Item Standard Deviation. 0.

## Data Availability

The datasets used and/or analyzed during the current study are available from the corresponding author upon reasonable request.

## Appendix A. Perceived Therapist’s Knowledge about Gender Identity Diversity Scale

[Escala de Percepción sobre el Conocimiento en Terapia de la Diversidad de Identidad de Género].

	**Totalmente en Desacuerdo** **(1)**	**Algo en Desacuerdo** **(2)**	**Neutral** **(3)**	**Algo de Acuerdo** **(4)**	**Totalmente** **de****Acuerdo** **(5)**
1. Mi terapeuta parecía entender que mi identidad de género no es una enfermedad	☐	☐	☐	☐	☐
2. Mi terapeuta entendía que el género es construido socialmente	☐	☐	☐	☐	☐
3. Mi terapeuta reconocía que la identidad de género y la orientación sexual son conceptos separados	☐	☐	☐	☐	☐
4. Mi terapeuta parecía conocer cómo los prejuicios de la sociedad afectan mi salud mental	☐	☐	☐	☐	☐
5. Mi terapeuta parecía conocer cómo los prejuicios de la sociedad afectan mi salud física	☐	☐	☐	☐	☐
6. Mi terapeuta reconocía el estigma que existe sobre mi comunidad	☐	☐	☐	☐	☐
7. Mi terapeuta tenía conocimiento sobre la violencia que podría experimentar por mi identidad de género	☐	☐	☐	☐	☐
8. Mi terapeuta reconocía las barreras institucionales que me encuentro debido a mi identidad de género	☐	☐	☐	☐	☐
9. Mi terapeuta entendía cómo mi identidad de género puede variar a través del tiempo	☐	☐	☐	☐	☐
10. Mi terapeuta parecía reconocer el estrés que me afecta por ser una minoría social	☐	☐	☐	☐	☐
11. Mi terapeuta tenía conocimiento de cómo las fuentes de apoyo eran necesarias para fortalecer mi salud mental	☐	☐	☐	☐	☐
12. Mi terapeuta parecía entender los retos de las relaciones románticas y sexuales que trae mi identidad de género	☐	☐	☐	☐	☐
Note: No hay ítems inversos. Esta escala es de libre uso. No necesita permiso de la autoría para ser utilizada. En caso de ser utilizada, modificada o adaptada, agradecemos que nos envíen los resultados y los cambios para nuestro conocimiento. The Scale was developed in Spanish. The authors do not have an English version of the scale.					
